# Training primary healthcare workers on a task-strengthening strategy for integrating hypertension management into HIV care in Nigeria: implementation strategies, knowledge uptake, and lessons learned

**DOI:** 10.1186/s12913-023-09603-4

**Published:** 2023-06-21

**Authors:** David Ayoola Oladele, Aina Olufemi Odusola, Oluwatosin Odubela, Ucheoma Nwaozuru, Colvin Calvin, Zaidat Musa, Ifeoma Idigbe, Chioma Nwakwo, Yemi Odejobi, Angela Aifah, Nafesa Kanneh, Shivani Mishra, Deborah Onakomaiya, Juliet Iwelunmor, Olugbenga Ogedegbe, Oliver Ezechi

**Affiliations:** 1 Nigerian Institute of Medical Research, Yaba, Lagos, Nigeria; 2 Department of Behavioral Science and Health Education, College for Public Health and Social Justice, Saint Louis University, Saint Louis, MO 63103 USA; 3 Lagos State University Teaching Hospital, Ikeja, Lagos, Nigeria; 4 Wake Forest School of Medicine, Winston-Salem, NC 27101 USA; 5 New York University School of Medicine, New York City, NY 10016 USA

**Keywords:** Task-strengthening, Hypertension, HIV, Healthcare workers, TASSH

## Abstract

**Background:**

With improved access to anti-retroviral drugs, persons living with HIV/AIDS (PLWHA) are living longer but with attendant increased risks of non-communicable diseases (NCDs). The increasing burden of NCDs, especially hypertension, could reverse gains attributed to HIV care. Nurses and Community Health Officers (CHO) in Nigeria are cardinal in delivering primary health care. A task-strengthening strategy could enable them to manage hypertension in HIV care settings. This study aimed to assess their knowledge and practice of hypertension management among Healthcare workers (HCWs) and to explore the challenges involved in conducting onsite training during pandemics.

**Methods:**

Nurses and CHOs in the employment of the Lagos State Primary Health Care Board (LSPHCB), Lagos State, Nigeria, were recruited. They were trained through hybrid (virtual and onsite) modules before study implementation and a series of refresher trainings. A pre-and post-training test survey was administered, followed by qualitative interviews to assess skills and knowledge uptake, the potential barriers and facilitators of task-sharing in hypertension management in HIV clinics, and the lessons learned.

**Results:**

Sixty HCWs participated in the two-day training at baseline. There was a significant improvement in the trainees' knowledge of hypertension management and control. The average score during the pre-test and post-test was 59% and 67.6%, respectively. While about 75% of the participants had a good knowledge of hypertension, its cause, symptoms, and management, 20% had moderate knowledge, and 5% had poor knowledge at baseline. There was also an increase in the mean score between the pre-test and post-test of the refresher training using paired t-tests (*P* < 0.05). Role-playing and multimedia video use improved the participants' uptake of the training. The primary barrier and facilitator of task sharing strategy in hypertension management reported were poor delineation of duties among HCWs and the existing task shifting at the Primary Healthcare Centres (PHC) level, respectively.

**Conclusions:**

The task strengthening strategy is relevant in managing hypertension in HIV clinics in Nigeria. The capacity development training for the nurses and CHOs involved in the Integration of Hypertension Management into HIV Care in Nigeria: A Task Strengthening Strategy (TASSH-Nigeria) study yielded the requisite improvement in knowledge uptake, which is a reassurance of the delivery of the project outcomes at the PHCs.

## Background

Hypertension remains a significant disease of public health concern, and it represents the major risk factor for cardiovascular diseases [1]. About a quarter of the world's adult population has hypertension, which is projected to increase to 29% by 2025 [2]. The prevalence of hypertension in sub-Saharan Africa (SSA) ranges from 25.9% to 46% [3, 4], and in Nigeria, the prevalence ranges from 25% to 30.6% [5]. SSA is also plagued with a high burden of infectious diseases like the Human Immunodeficiency Virus and Acquired Immune Deficiency Syndrome (HIV/AIDS). About 70% of the persons living with HIV/AIDS (PLWHA) live in SSA. Nigeria is second only to South Africa in the HIV burden on the continent [6].

With increased access to life-saving antiretroviral medications and its scale-up, PLWHA are living longer and, as a result, have an increased risk of developing non-communicable diseases (NCDs), especially hypertension [7]. In Nigeria, there has been a report of the increased burden of cardiovascular diseases, including hypertension, among PLWHA. The prevalence of hypertension from two regional HIV clinics was reported to be between 17 and 31% [8, 9]. This implies that countries in SSA are at increased risk of the double burden of infectious and non-communicable diseases. Therefore, integration of hypertension care into HIV clinics is necessary to prevent morbidity and mortality from NCDs amongst PLWHA.

Aside from the double burden of infectious diseases and NCDs, countries in SSA are also faced with challenges of shortage of competent health workforce mainly because of the increasing population, fewer training facilities, and increasing migration of the few trained professionals for greener pastures abroad [10]. To limit the effect of healthcare human resource shortage, the World Health Organization (WHO) has proposed the evidence-based practice of the task-shifting strategy [11]. Task shifting strategy, according to the WHO, "involves the rational redistribution of tasks among health workforce teams. Specific tasks are moved, where appropriate, from highly qualified health workers to health workers with shorter training and fewer qualifications to make more efficient use of the available human resources for health" [11]. This method has been used to promote the scale-up of HIV care in SSA with great success and the provision of care, especially at Primary Healthcare Centres (PHC) and health posts in communities [12]. Furthermore, using the task-shifting strategy in treating hypertension at healthcare clinics has been shown to be effective [13]. However, the task-strengthening strategy is employed in this study because the task-shifting strategy is currently being implemented in Lagos State PHCs.

The Integration of Hypertension Management into HIV Care in Nigeria: A Task-Strengthening Strategy (TASSH-Nigeria) project is a cluster randomized controlled clinical trial. The project is aimed at evaluating through a hybrid clinical effectiveness-implementation design the effect of a replicable practice facilitation (PF) strategy to implement an evidence-based Task-Strengthening Strategy for Hypertension control (TASSH) as an integrated model for people living with HIV (PLWHA) across 30 primary healthcare centers (PHCs) in Lagos, Nigeria [14].

The importance of training in developing the health workforce's capacity to deliver evidence-based practice cannot be overemphasized. This is because training and retraining are essential to developing a workforce to meet existing needs [15]. Many methods can be utilized in the conduct of this training/education. This can include group education, personalized education, usually through precepting/on-the-job training, and incorporation of learning through the internet or a hybrid of in-person and virtual learning [16]. It has been documented that after a successful initial education, a retraining process is essential to ensure the sustainability of the knowledge gained and the skills acquired. Though continuing education in the work environment has its challenges, training, and retraining have been documented to result in better patient safety and improvement in professionals' in-depth knowledge and overall better-quality patient care [17].

This study aims to document the knowledge uptake and capacity building of the healthcare workers (Nurses and CHOs) involved in the delivery of the TASSH project at the PHCs and to describe some challenges encountered and lessons learned in the process.

## Methods

Based on a mixed method study design, we describe TASSH training for nurses and CHOs in Lagos State, Nigeria.

### Context

The study took place in Lagos state, a cosmopolitan state in Nigeria, and the country's commercial nerve center. Lagos has 20 local government areas (LGAs), of which 16 are classified as urban and the remaining four as rural. Lagos State is located in the South-West geopolitical zone of Nigeria. It is the smallest state in Nigeria but with the largest urban population, 27.4% of the national estimate. According to the UN-Habitat and international development assessments, Lagos State had about 24.6 million inhabitants in 2015 [18]. Also, the state adopts primary healthcare as the central hub of its healthcare delivery system. The Lagos State Primary Healthcare Board (LSPHCB) manages about 100 community health centers with over 67 PHCs with a focus on community health and the strengthening of human resources for integrated health services. LSPHCB works with the Lagos State AIDS Control Agency (LSACA) to provide comprehensive HIV care across its 67 PHCs.

The TASSH-Nigeria project implementation phase involved the development of the components of the Practice Facilitation strategy. This research project is a collaborative effort between New York University School of Medicine, NYU Langone Health, USA; Saint Louis University, Missouri, USA; and the Nigerian Institute of Medical Research, Lagos, Nigeria (PROTOCOL NUMBER: NCT Number (NCT04704336), Other Study ID (20–00009); US NIH Grant/ Contract (R01HL147811-01A1). The protocol for the TASSH-Nigeria project that documented the objectives and phases of implementation has been published elsewhere [14].

Healthcare workers (HCWs) to be involved in the delivery of the TASSH intervention at the PHCs were invited through the Lagos State Primary Healthcare Board (LSPHB) to a two-day pre-commencement training and subsequently refresher trainings to reinforce their understanding and practice of the project protocol and activities. Two HCWs from the identified PHCs participated in the pre-commencement training. A total of 30 PHCs providing comprehensive HIV care to PLWHA were randomly assigned to either the PF intervention (*N =* 15) or the self-directed condition (*N =* 15).

The TASSH-Nigeria team from the Nigerian Institute of Medical Research (NIMR) worked through the existing long history of collaborative engagement and partnership with the Lagos State Primary Healthcare Board (LSPHCB) and Lagos State AIDS Control Agency (LSACA) to train the HCWs and facilitate recruitment of participants for the study.

### Training content/procedures

The baseline training involved 60 HCWs (30 Nurses and 30 CHOs) invited from all the 30 PHCs selected for the study. The training took place at the auditorium of NIMR for two days. A series of refresher trainings were conducted at intervals to reinforce the skills of the HCW at the point which coincides with the onboarding of the PHCs into the project.

The training methodology involved didactic lectures, hands-on training, multimedia video, and discussion to suit the adult learning methodology. The baseline training was done through virtual and in-person training with facilitators from NYU, NIMR, and Lagos State University Teaching Hospital. In this study, we reported on the baseline training done in April 2021 and refresher training done in February, May, and August 2022.

Specifically, participants were trained on the TASSH-Nigeria study protocol, hypertension fundamentals, and hypertension management using the Nigeria treatment guidelines for hypertension. They were also trained on hands-on clinical measurements of blood pressure, height, weight, hip and waist circumference, and arm circumference. A role-play practice session on clinical measures was done. Participants also had training on the 5 A's of counseling patients with a diagnosis of hypertension which involved: Ask-asking the patients about the knowledge of their blood pressure status; Assess – assessing the patient's comprehension of their high blood pressure status and diagnosis; Advise-advising the patients on going to the clinic visit; Assist-assisting the patient to understand how to access care in the clinic; and Arrange- scheduling the patient's follow-up visit at the clinic. A Multimedia video on the 5 A's adapted to the Nigerian context was used to reinforce the understanding of the HCWs on best practices for communicating with patients during a clinical encounter at a health facility.

An adapted questionnaire was used to assess the knowledge of the HCWs on the symptoms, diagnosis, and treatment of hypertension [19]. Also, some basic information about the study procedure and expected activities at the PHCs was assessed through a pre-test and post-test. Additionally, an interview guide was used to conduct exit interviews among the study participants to understand better their uptake of the training and the perceived barriers and facilitators of implementing the evidence-based intervention at their PHCs.

### Data management and analysis

Data analysis was done using IBM SPSS version 26. Discrete variables are presented as percentages, while continuous variables were expressed as means (± standard deviation). The categorization of hypertension knowledge was based on the percentage score during the pre-test. Those who scored 80% and above were said to have good knowledge, those who scored 65% to 79% were categorized as average knowledge, and those who scored less than 65% were classified as poor. Proportions were compared using Pearson Chi-square, and fishers exact for categorical variables. The paired student's t-test was used to compare the mean difference in the pre-test and post-test scores for all the studies. The level of significance was predetermined at *p* < 0.05. The interviews and reflections from the training participants were digitally recorded, the recordings were transcribed verbatim, and thematic analysis was used to produce emerging themes.

## Results

The baseline training was attended by 60 HCWs (30 nurses and 30 CHOs) from the selected thirty PHCs in Lagos state. The mean age of the participants was 41.4 ± 9.5 years, and they were mostly females (93.3%). Most participants were married (81.7%) and a significant proportion had diplomas (50%) with an average of 15.4 (± 9.7) years of professional practice (Table 1).

**Table 1:** Baseline sociodemographic characteristics of the participants in the training

**Variable**	**Frequency(** ***N =*** ** 60)**	**Percentage**
Occupation		
CHO	30	50
Nurse	30	50
Age	Mean* =* 41.4±9.5	
Gender		
Female	56	93.3
Male	4	6.7
Education		
Diploma	30	50
BSc	26	43.3
MSc	4	6.7
Religion		
Christianity	48	80
Islam	12	20
Marital Status		
Married	49	81.7
Single	11	18.3
How many years of practicing	Mean* =* 15.4±9.7	
Years of education	Mean* =* 12±9.7	

Overall, 75% of the participants had a good understanding of the knowledge, cause, symptoms, and management of hypertension, while 20% and 5% of participants had moderate and poor knowledge, respectively (Table 2).

**Table 2:** Baseline knowledge of participants about hypertension

**Variable**^**a**^	**Frequency**	**Percentage**
Good knowledge	45	75
Average knowledge	12	20
Poor knowledge	3	5

^a^Good Knowledge (scored 80% and above in pre-test), Average Knowledge (scored 65–79% in pre-test) and Poor Knowledge (scored less than 65% in pre-test)

The average pre-test and post-test score was 59% and 67% respectively during the baseline training. There was a significant improvement in the baseline knowledge of participants on hypertension management among HIV-positive patients as well as their understanding of the TASSH study protocol and processes (*p* =  < 0.01) (pre-test (Mea*N =* 17.8, SD = 3.31) and post-test (Mea*N =* 20.3, SD = 2.91) respectively). Also, there was an improvement in the mean score of the pre-test and post-test during the refresher trainings (Table 3).

**Table 3:** Paired sample t-test results for pre-and post-training measures of knowledge at each training

**Training schedules**	**Mean**	**Standard Deviation**	**Paired** **t-test**	**df**	**Significant** **2-tailed**
Baseline pre-test	17.8	3.31	-5.04	57	< 0.01
Baseline post-test	20.3	2.91			
1^st^ refresher training pre-test	22.3	2.77	-4.27	25	< 0.01
1^st^ refresher training post-test	24.1	2.62			
2^nd^ refresher training pre-test	23.5	3.6	-1.97	19	0.06
2^nd^ refresher training post-test	25.1	2.67			
3^rd^ refresher training pre-test	19.5	2.23	-3.05	19	< 0.01
3^rd^ refresher training post-test	26.6	10.3			

**Fig. 1:**
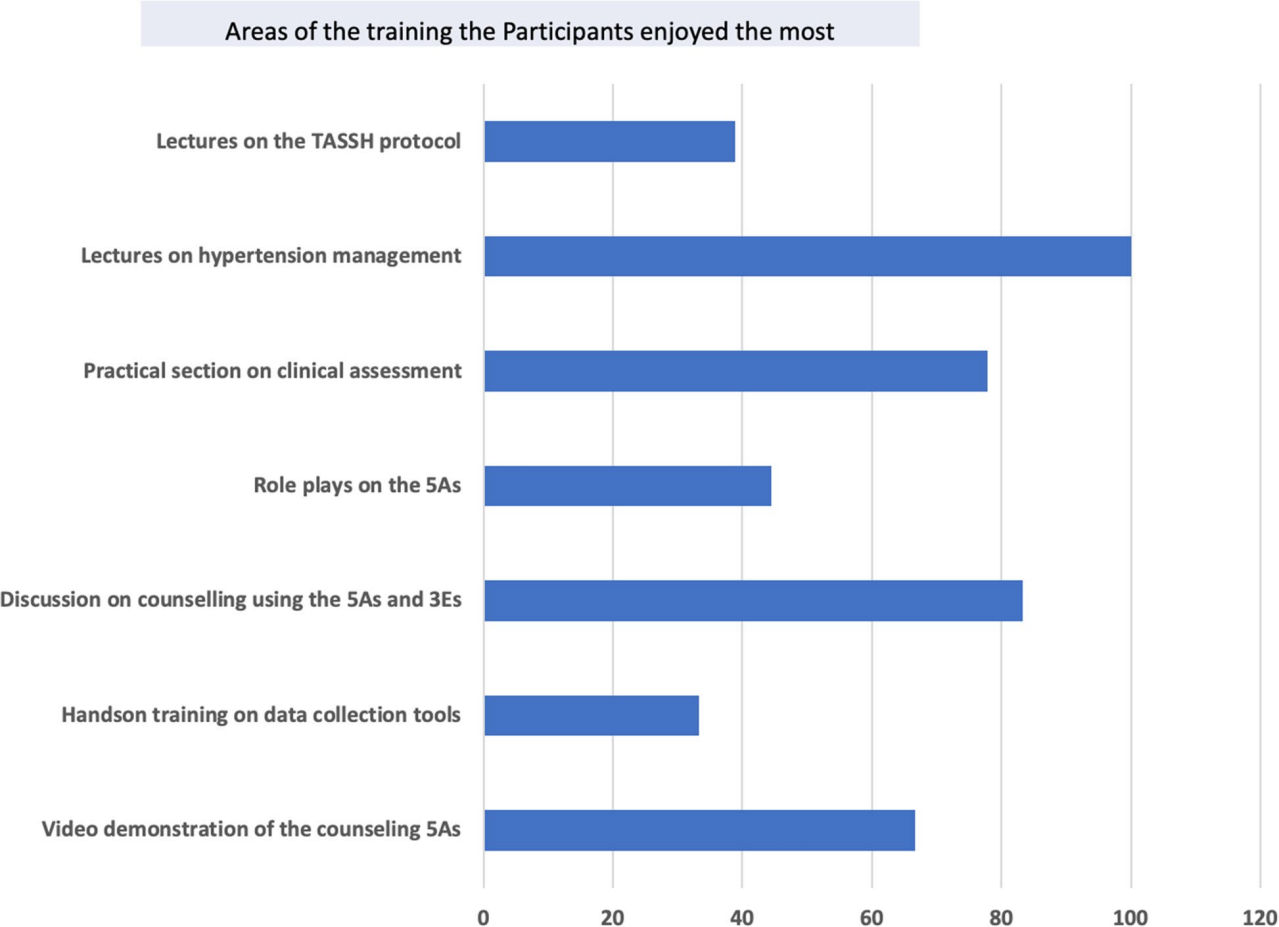
Participant's feedback on the training uptake

The participants' reflections after the training showed that all the participants enjoyed the lecture on hypertension management (100%) and discussions on counseling patients with hypertension using the 5 A's. The aspect of the training they least enjoyed was the hands-on training on the data collection tools (Fig. 1).

**Fig. 2:**
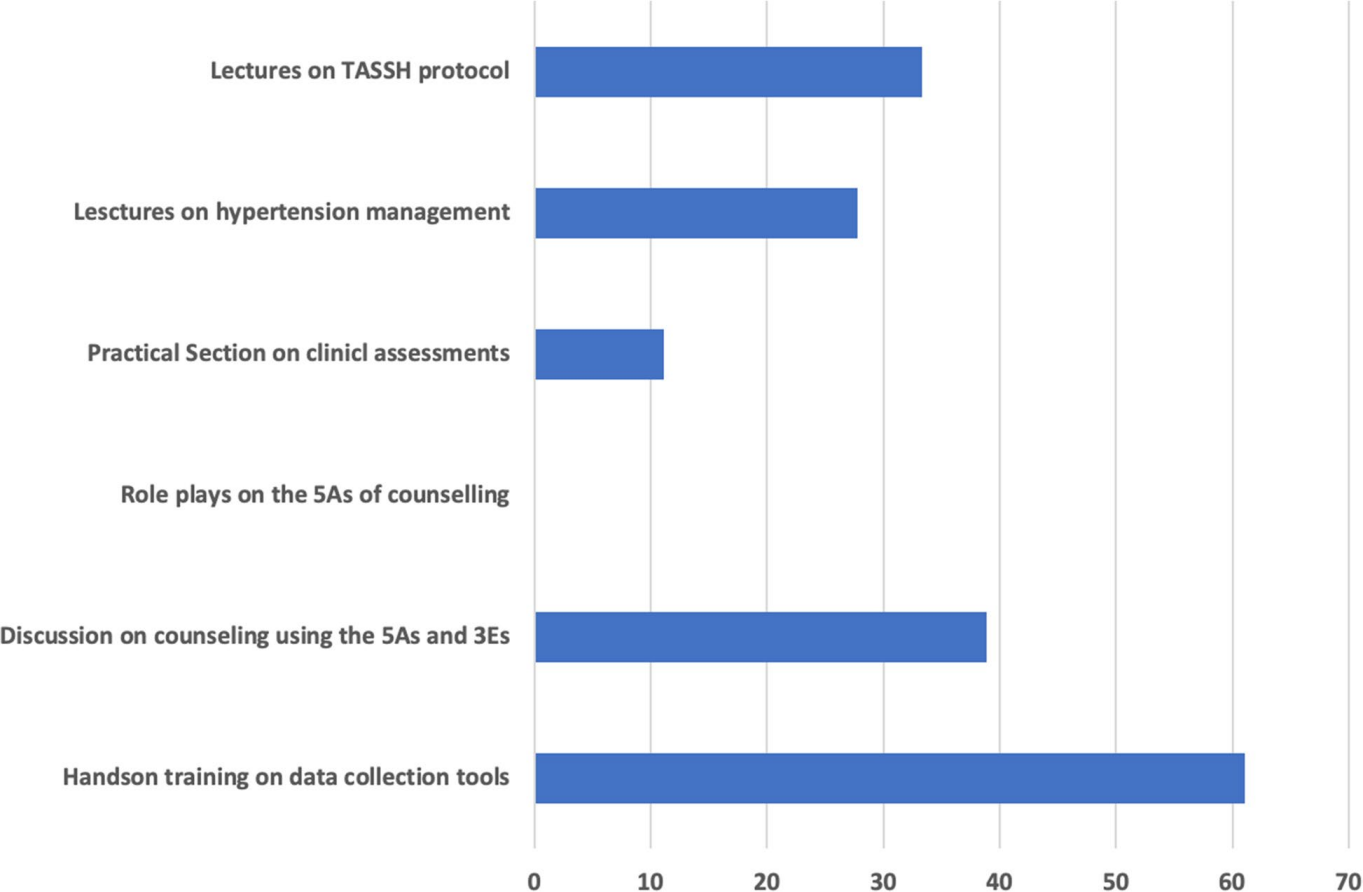
Participant's feedback on the areas of need for future training

The participants also submitted that they would need more emphasis on the hands-on practice with the data collection tools (61.1%) as well as discussion on the 5As (38.9%) and lectures on the TASSH study protocol (33.3%) (Fig. 2).

**Fig. 3:**
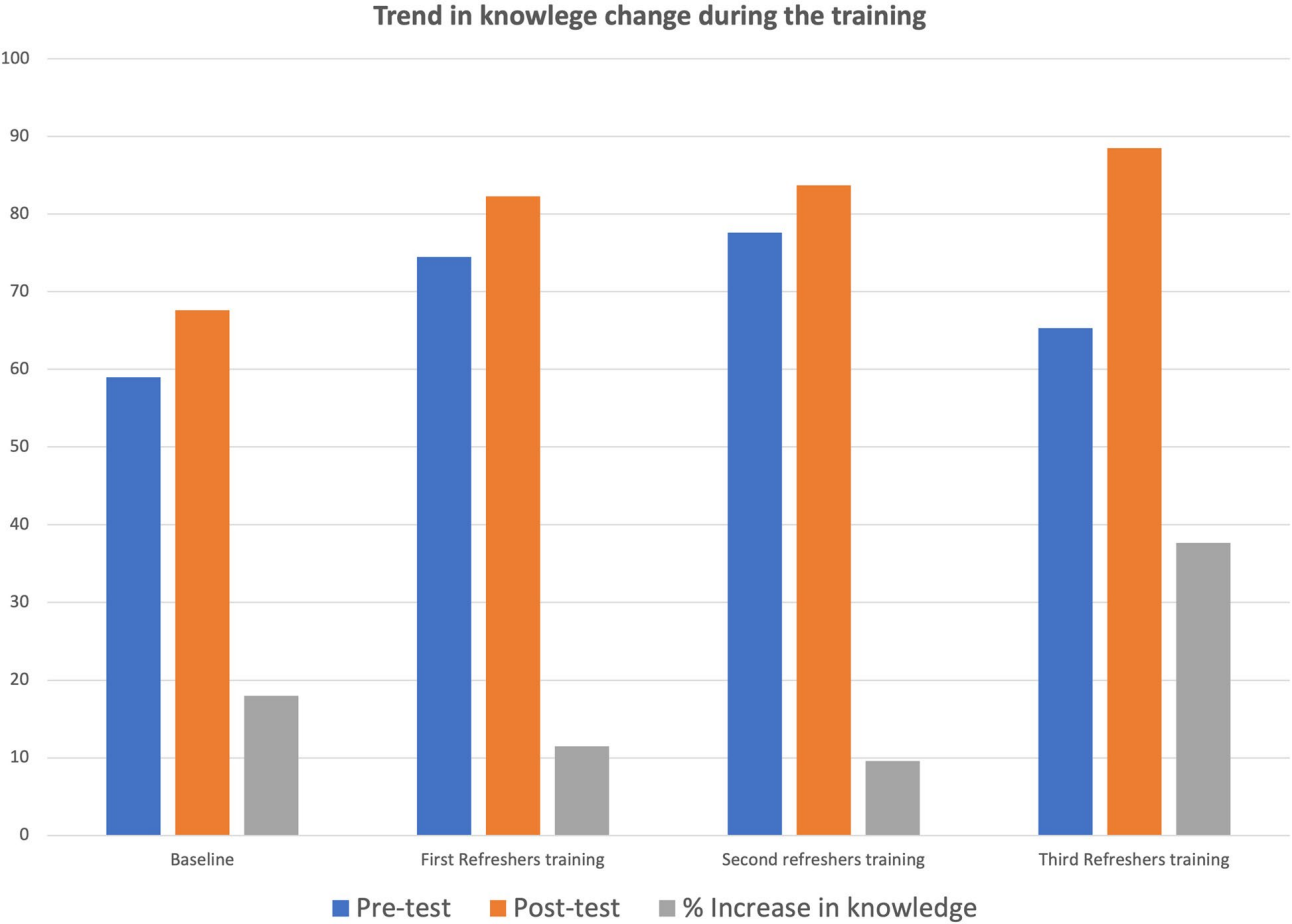
Trend in knowledge change among participants at TASSH training

Furthermore, we compared the outcome of the subsequent three trainings for the Nurses and CHOs. Figure 3 reported the percentage increase in knowledge acquisition trend during the four training interactions. There was an increase in knowledge acquisition during the trainings, with the most significant increase observed in the third refresher training. Also, comparing the pre-test and post-test of these training showed an increase in the mean score between the pre-test and post-test using paired t-tests. However, the mean difference between the pre-test and post-test of the second refresher training was not statistically significant (Table 3).

### Qualitative result

The participants reflected that they were confident in implementing the learned components of the TASSH study at their PHCs with little or no supervision. However, some observed that the major challenge might be the increased workload due to the perennial staff shortage.

"It is good to come for a training like this, but to carry it out at the facility is difficult. For me, when I resume work, I do health talks, counsel pregnant women, and palpate the abdomen of women in Labour, and it's only me too that will attend to the HIV patient. It is not easy."

Also, it was observed that HIV patients come to the clinic for different purposes, including drug refills, clinic consultations, and laboratory assessments. There is a need to put structures in place to ensure that every PLWHA coming to the clinic sees the trained staff for hypertension screening.

"if care is not taken, we might not be able to screen the patients when they come because we might not see them if they only came to collect their HIV drugs at the pharmacy."

The exit interviews also revealed that possible challenges for implementing the TASSH project include poor delineation of duties and frequent staff transfer. At the same time, the existing task-shifting policy at the PHCs could be a facilitator of task-sharing strategy in hypertension management in HIV in Lagos state.

Challenges were encountered in conducting the capacity development training for the HCWs on the TASSH project. First, mobilizing the HCWs to attend the training after an invitation was a challenge mainly because of the demands on their work and conflicting duty schedules. It was necessary to address this adjustment to accommodate all the invited HCWs and actively follow up with their supervising officers to allow timely release for future trainings. Also, many HCWs presenting for the refresher trainings were not part of the initially trained cohort due to frequent inter-facility transfers and emigration outside the country. This justifies the adjustment to make the refresher training more frequent, encouraging HCWs to conduct step-down training at their facilities/PHCs, and periodic engagement of the Nurses/CHO through the WhatsApp platform to remind them of key concepts about the project. The administrative challenge of engaging with the LSPHCB about releasing the HCWs to participate in the training and those posed by their limited availability from involvement in the state-wide COVID-19 vaccine rollout was addressed through sustained advocacy visits and engagement with State officials (Table 4).

**Table 4:** Challenges and lessons learned in the implementation of the TASSH training

	**Challenges**	**Description**	**Lessons Learned**
1	Mobilization and engagement of trainees	This is due to few staff at the PHCs and the conflicting clinical duties of the HCWs. A significant proportion came late to the training venue	• The need to liaise with clinic head through their Medical Officers of Health for the release of the HCWs • Communication of the date of training on time to give enough time for planning • Need for adjustment of the time of the training to accommodate late comers
2	HCWs attrition and inter-facility transfers	Many of the HCWs trained during the baseline have left the facility because of frequent staff turn-around secondary to emigration and inter-facility transfers	• Conduct of frequent refresher training is necessary to close the knowledge gap • Study research Nurse assesses and conduct onsite hands-on training • Step down training of TASSH study to other CHO/Nurses at the facility
3	Administrative challenges of engaging Government Agencies	There was difficulty in agreeing on the date for the training because of conflicting programs of the Lagos State PHCB that involves the same set of HCWs	• The need for continued engagement of the TASSH-Nigeria team with the leadership of the LSPHCB • Early initiation of the request for approval and invitation of the HCWs is crucial • Cooperative agreement on the TASSH calendar and LSPHCB is crucial
4	COVID-19 Vaccine roll out in Nigeria	There was difficulty in inviting the HCWs for training during the rollout of the COVID-19 vaccine in Lagos	• All the HCWs were directly involved with the vaccination exercise. Hence the study process has to be adjusted to accommodate their schedule • Adaptation of the implementation of the study timeline is necessary based on the realities of the implementation climate

## Discussion

We describe the strategies, knowledge uptake, and lessons learnt in the training of HCWs involved in the TASSH-Nigeria project. The high burden of hypertension is a primary concern in Nigeria, just as in other parts of SSA, especially since PLWHA are living longer with improved quality of life [20, 21]. The main thrust of the trainings were to provide nurses and CHOs with requisite skills to deliver the TASSH project at their PHCs and build local and institutional capacity for the management of hypertension among PLWHA in primary care settings.

From the study findings, we were able to demonstrate that a study that employs a variety of training modules (lectures, multimedia video presentations, discussions, and hands-on clinical experience) improved the knowledge uptake of HCWs working at the PHCs level in Lagos, Nigeria. This finding further corroborates the opinion expressed by previous trainings among HCWs that suggest a hybrid model of training being the best model for training busy HCWs in developing countries [22–24].

The knowledge uptake observed among the study participants was good as there was an increase in the mean score for the pre-test and post-test for the different iterations of training. However, the mean difference for the third training was not statistically significant. The observed significant difference in the percentage increase in knowledge for the fourth training was because the mean pre-test score was lower than those obtainable for the two previous tests, which could be secondary to the fact that many of the participants at the training were undergoing the training for the first time. Other studies in SSA documented frequent attrition and transfer of HCWs, especially after the COVID-19 lockdown period [25, 26]. The participants’ reflection on the areas of emphasis for future training engagement suggests that they were able to make an objective evaluation of their skill acquisition during the TASSH training sessions. This was a positive takeaway from the training, thus aiding the planning process for subsequent trainings.

Our study found that the mean age (± SD) of the HCWs was 41 (± 9.5) years, and the mean years (± SD) of work experience were 15.95 (± 9.7) years. This suggests that the HCWs involved in the study have the requisite expertise and emotional competence, making them ideal candidates for implementing task-shifting/strengthening programs at the PHCs. Also, they are most suited to deliver on the expectations of the training because of their expertise/competence. This is because years of experience was reported to be correlated with emotional competence [27] and individuals with more years of experience were less likely to report job-related stress [28]. Moreover, their baseline knowledge of hypertension was found to be adequate, as about 75% had good knowledge of hypertension. This is similar to the study reported in Uganda in which HCWs had considerable good knowledge of hypertension [29].

As submitted by participants in the qualitative interviews, acute shortage of the needed HCWs at the HIV clinics and work overload of the few available ones are a reality in SSA settings. The suggestion that government agencies should engage more hands and improve the organization of tasks at the healthcare centers seems appropriate and pragmatic because addressing human resources for health challenges in SSA is a major concern [30, 31]. Most of the challenges encountered in executing the TASSH training program are not unexpected in developing country settings [32], and the lesson learnt in the process is invaluable to the TASSH project team in the planning for future trainings. It could also be a reference material for other groups planning training in similar settings.

One of the study's strengths is that we reported pragmatic findings encountered in implementing a novel evidence-based intervention in Nigeria. This has the potential to define how the care of hypertension at HIV clinics at the primary healthcare level is done and how the capacity of HCWs in these settings can be improved. In addition, employing the qualitative component to explore the perception of the training will further assist in situating the success, challenges, and potential for improving subsequent trainings.

A significant limitation of the reported study is the fact that this is descriptive in nature with a small population of HCWs. Hence, we cannot establish causality, and the findings might have limited strength of generalizability. Also, some of the responses from the qualitative studies could be subject to social desirability bias among the HCWS. Another limitation is the fact that the frequent transfer and possible attrition of the health workers trained at baseline could constitute a challenge in the determination of improvement in knowledge of trained HCWs. Hence, the reported change in knowledge in this study should be interpreted in this light.

The results of this study indicate that continuous capacity-building training of HCWs can result in an improvement in specific skills and delivery of an evidence-based intervention. Also, with HCWs' satisfaction with training programs, there is likely to be improved self-efficacy and professional competence to deliver the requisite care. Furthermore, a hybrid mode of training is likely to improve knowledge uptake and document what works, especially in adapting to the challenge of the COVID-19 pandemic. Moreso, challenges encountered in HCWs training would enhance the assessment of training needs and the conduct of future trainings. To address the challenge of frequent staff transfers and turnover, step-down training at the facility level for all nurses and CHO on the diagnosis and management of hypertension would be recommended for the regulatory body.

## Conclusion

The Task-sharing strategy is relevant in managing hypertension in HIV clinics in Nigeria. The capacity development training for the Nurses and CHO involved in the TASSH-Nigeria study yielded the requisite improvement in knowledge uptake, which is a reassurance of the delivery of the project outcomes at the PHCs. The need for sustained engagement with state level healthcare managers towards the delivery of the study is critical. Continued refresher trainings for HCWs is necessary to address the challenge of staff attrition and movement between health facilities. Overall, the study highlighted a considerable amount of well-documented insights at the facility level and HCWs that could improve service delivery at the PHCs and meet the health needs of PLWHA in Nigeria.

## Data Availability

The datasets used and/or analyzed during the current study are available from the corresponding author upon reasonable request.

## Abbreviations

HIV/AIDS: Human Immunodeficiency Virus/Acquired Immune Deficiency Syndrome
PLWHA: Persons living with HIV/AIDS
CHO: Community Health Officers
LSPHCB: Lagos State Primary Health Care Board
LSACA: Lagos State AIDS Control Agency
NIMR: Nigerian Institute of Medical Research
NYU: New York University School of Medicine, NYU Langone Health
HCWs: Healthcare workers
PHCs: Primary Healthcare Centres
TASSH: Task-strengthening strategy for integrating hypertension management into HIV care in Nigeria
SSA: Sub-Saharan Africa
NCD: Non-communicable diseases
HTN: Hypertension
WHO: World Health Organization
CFIR: Consolidated Framework for Implementation Research
RE-AIM: Reach, Effectiveness, Adoption, Implementation, and Maintenance Framework
